# The Association Between Dietary Inflammatory Index and Parathyroid Hormone in Adults With/Without Chronic Kidney Disease

**DOI:** 10.3389/fnut.2021.688369

**Published:** 2021-06-25

**Authors:** Zheng Qin, Qinbo Yang, Ruoxi Liao, Baihai Su

**Affiliations:** Department of Nephrology, National Clinical Research Center for Geriatrics, West China Hospital, Sichuan University, Chengdu, China

**Keywords:** dietary inflammatory index, parathyroid hormone, chronic kidney disease, hyperparathyroidism, National Health and Nutrition Examination Survery

## Abstract

**Aims:** We aimed to assess the association between dietary inflammation index (DII) with parathyroid hormone (PTH) and hyperparathyroidism (HP) in adults with/without chronic kidney disease (CKD).

**Methods:** Data were obtained from the 2003–2006 National Health and Nutrition Examination Survey (NHANES). The participants who were <18 years old, pregnant, or missing the data of DII, PTH, and CKD were excluded. DII was calculated based on a 24-h dietary recall interview for each participant. Weighted multivariable regression analysis and subgroup analysis were conducted to estimate the independent relationship between DII with PTH and the HP in the population with CKD/non-CKD.

**Results:** A total of 7,679 participants were included with the median DII of −0.24 (−2.20 to 1.80) and a mean PTH level of 43.42 ± 23.21 pg/ml. The average PTH was 45.53 ± 26.63 pg/ml for the participants in the highest tertile group compared with 41.42 ± 19.74 pg/ml in the lowest tertile group (*P* < 0.0001). The rate of HP was 11.15% overall, while the rate in the highest DII tertile was 13.28 and 8.60% in the lowest DII tertile (*P* < 0.0001). The participants with CKD tended to have higher PTH levels compared with their counterparts (61.23 ± 45.62 vs. 41.80 ± 19.16 pg/ml, *P* < 0.0001). A positive association between DII scores and PTH was observed (β = 0.46, 95% CI: 0.25, 0.66, *P* ≤ 0.0001), and higher DII was associated with an increased risk of HP (OR = 1.05, 95% CI: 1.02, 1.08, *P* = 0.0023). The results from subgroup analysis indicated that this association was similar in the participants with different renal function, gender, age, BMI, hypertension, and diabetes statuses and could also be appropriate for the population with CKD.

**Conclusions:** Higher consumption of a pro-inflammatory diet appeared to cause a higher PTH level and an increased risk of HP. Anti-inflammatory dietary management may be beneficial to reduce the risk of HP both in the population with and without CKD.

## Introduction

Parathyroid hormone (PTH) is a single-stranded peptide hormone, containing 84 amino acids, which are synthesized and secreted by the chief cells of the parathyroid gland, with the main function of increasing the serum Ca^2+^ and decreasing the serum phosphorus levels (1). The secretion of PTH is also mainly regulated by th zvbe concentration of serum Ca^2+^ and phosphorus (2, 3). Serum Ca^2+^ regulates PTH secretion through the interaction with calcium-sensitive receptors (CASR) on the surface of parathyroid cells (4, 5). Serum phosphorus enhances the stability of PTH mRNA and stimulates the proliferation of parathyroid cells to increase the secretion of PTH both directly and indirectly (3, 6). Bone and kidney are the main target organs of PTH (7–9). For a variety of pathological reasons, the parathyroid glands can secrete excessive PTH and cause hyperparathyroidism (HP), which can be classified as primary, secondary, and tertiary (10). HP appeared to be associated with an increased risk of poor clinical outcomes and death, which is often observed in patients with chronic kidney disease (CKD) (11).

Chronic kidney disease refers to a chronic clinical condition of renal, structural, and functional disorders and is characterized by a higher inflammation status (12). Recent studies have revealed the global prevalence of CKD to be about 10%, as well as the increasing disease burdens (13–15). In patients with CKD, abnormal regulation in calcium, phosphorus, vitamin D, and PTH is accompanied by a decline in renal function, which could lead to secondary HP, which is associated with the increased risk of fracture, cardiovascular disease, and death (16–18). Thus, the management of the PTH level in patients with CKD is of great significance.

The association between inflammation and PTH remains unclear. Several animal and human studies indicated that the PTH level may be associated with inflammation (19–21). Previous studies observed a decreased inflammation status after parathyroidectomy in patients with HP (22, 23). However, the inflammation level after parathyroidectomy varies widely among studies; both increased (24) and even no-change results (25) have been reported before. Chen et al. (19) found that inflammatory markers, including C-reactive protein (CRP), red cell distribution width (RDW), and platelet-to-lymphocyte ratio (PLR) levels increased with increasing serum PTH concentration in the US adults, indicating a positive relationship between inflammation and PTH. In *in vitro* studies, several inflammatory cytokines, such as interleukin-8 (IL-8), tumor necrosis factor-α (TNF-α), etc., have been proved to enhance PTH synthesis and secretion through nuclear factor-κB (NF-κB) and affect CASR transcription (26–28). It could be inferred that the consumption of an inflammatory diet may also have an impact on the PTH. Dietary inflammatory index (DII) was a literature-derived and population-based scoring system designed to evaluate the inflammatory potential of diets (29). A positive value for DII corresponded to an pro-inflammatory diet, and a negative value for DII corresponded to an anti-inflammatory diet. The higher scores suggested a more pro-inflammatory effect, and the more negative scores suggested a more anti-inflammatory effect. In fact, previous studies indicated a significant association between DII and the risk of various cancers (30, 31), obesity (32), cardiovascular disease (33), sarcopenia (34), and so on. However, the association between the dietary inflammatory potential and PTH has not been reported before.

In this study, we aimed to assess the impact of DII on PTH and HP, using data from the National Health and Nutrition Examination Survey (NHANES). We hypothesized that the increased consumption of pro-inflammatory diet would be associated with higher PTH levels and increased risk of HP. In addition, with regard to the fact that patients with CKD may have more elevated PTH levels than the population with non-CKD, we further examined this association in subgroups stratified by renal function.

## Materials and Methods

### Study Population

In this study, we obtained data from the NHANES. NHANES is a program of studies administered by the National Center for Health Statistics (NCHS), part of the U.S. Centers for Disease Control and Prevention (CDC), aimed to assess the health and nutrition status of the U.S. population through interviews, physical examinations, and laboratory tests. The NHANES is conducted on a 2-year cycle, and the data are still being updated. Because this study adopted a stratified multistage probability sampling method, the included samples had good representativeness (35). All NHANES data are publicly available at www.cdc.gov/nchs/nhanes/.

**Figure 1:**
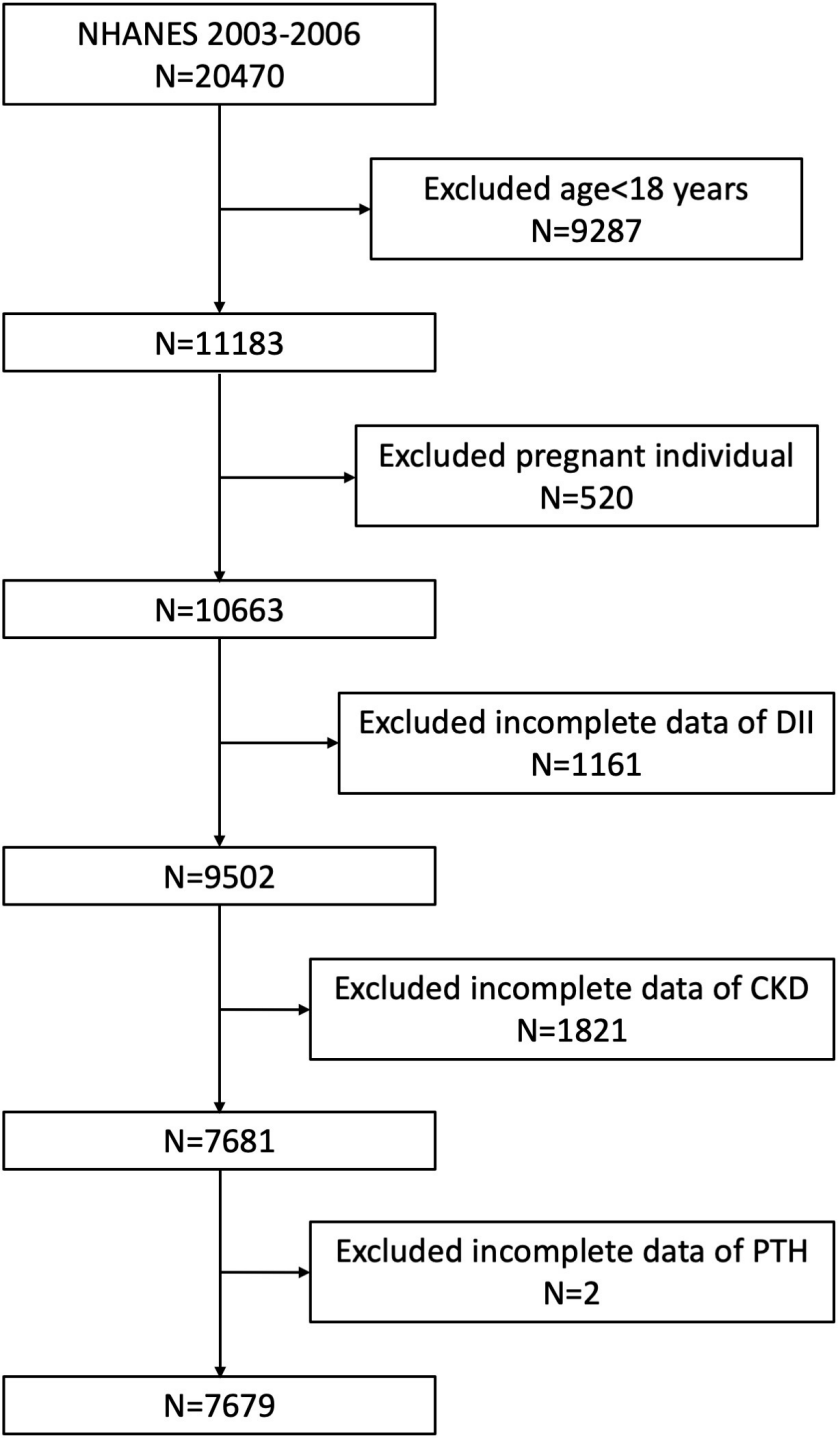
A flowchart of the sample selection from NHANES 2003–2006.

This study was based on the data from two 2-year NHANES surveys from 2003 to 2006. A total of 20,470 participants were enrolled at first; after the exclusion of individuals aged <18 years old (*n* = 9,287), who were pregnant (*n* = 520), missing the dietary data relating to DII (*n* = 1,161), missing the data of diagnosing CKD (*n* = 1,821), and missing the data of PTH (*n* = 2), 7,679 participants were included in our final analysis (Figure 1).

The NCHS Ethics Review Board granted the human subject approval for the conduction of NHANES, and written informed consent was obtained from all the participants.

### Exposure and Outcome Definitions

Dietary inflammation index was designed as an exposure variable. The dietary data in NHANES were obtained by a 24-h dietary recall interview at the mobile examination center (MEC) (36), which have been validated by the Nutrition Methodology Working Group before (37). The data of 24-h dietary recall interviews were used to calculate the DII scores for each individual, and it could quantify the inflammatory potential of diets. A higher positive DII score indicated a pro-inflammatory diet, and a lower negative DII score indicated an anti-inflammatory diet (29). A total of 27 food parameters were available in NHANES and were used for the calculation of DII, including anti-inflammatory food parameters (alcohol, β-carotene, fibers, folic acid, magnesium, zinc, selenium, vitamin A, vitamin B-6, vitamin C, vitamin E, monounsaturated fatty acid, niacin, riboflavin, n-3 fatty acid, n-6 fatty acid, polyunsaturated fatty acid, caffeine, and thiamin), and pro-inflammatory food parameters (cholesterol, carbohydrates, energy, fats, iron, vitamin B-12, protein, and saturated fat). Studies have shown that the predictive ability was not affected when <30 dietary parameters were used to calculate DII scores (38–40). DII was analyzed as a continuous variable, and the participants were divided into tertiles from the total sample for further analysis.

The PTH level and HP were designed as outcome variables. The Elecsys 1010 analyzer (Roche Diagnostics) was used to determine the serum intact PTH level. The Elecsys 1010 analyzer was a fully automatic run-oriented analyzer system for the determination of immunological tests, using the ECL/Origen electrochemiluminescent process. All components and reagents for routine analysis were integrated into the analyzer. PTH was measured on the Elecsys 1010, using a sandwich principle. There was no difference in the equipment, lab method, or lab site between the combined two survey cycles in our analysis. HP was defined as PTH > 65 pg/ml according to previous studies (1, 41). The detailed process of measuring PTH was available at www.cdc.gov/nchs/nhanes/.

### Other Study Variables

Baseline variables in this study included age (years), gender, race, educational level, systolic blood pressure (mmHg), diastolic blood pressure (mmHg), body mass index (kg/m^2^), serum glucose (mg/dl), serum phosphorus (mg/dl), serum iron (ug/dl), serum CRP (mg/dl), serum 25 (OH) D (nmol/L), serum calcium (mg/dl), urinary creatinine (mg/dl), urinary albumin (mg/L), urinary creatinine (mg/dl), eGFR (ml/min/1.73 m^2^), parathyroid hormone (pg/ml), hypertension, diabetes, albuminuria, low eGFR, and CKD. All detailed measurement processes of study variables were publicly available at www.cdc.gov/nchs/nhanes/.

We calculated urinary albumin: creatinine ratio (ACR) and defined albuminuria as ACR > 30 mg/g. The data about gender, age, and serum creatinine were used to calculate the estimated glomerular filtration rate (eGFR, ml/min/1.73 m^2^) according to the CKD Epidemiology Collaboration equation for each participant (42), and eGFR lower than 60 ml/min/1.73 m^2^ was used to define low eGFR. We defined CKD as the presence of either albuminuria or low eGFR according to Kidney Disease: Improving Global Outcomes 2012 recommendations (12).

### Statistical Analysis

All statistical analyses were conducted according to CDC guidelines (43). All estimates were calculated, accounting for NHANES sample weight. Continuous variables were presented as mean with SD or a median with an interquartile range, and categorical variables were presented as frequency or percentage. Either weighted Student's *t*-test (for continuous variables) or weighted chi-square test (for categorical variables) were conducted to calculate the differences in different DII groups (tertiles). To examine the association between DII and PTH levels, weighted multivariable linear regression explored PTH as a continuous variable, and weighted multivariable logistic regression for HP was used as a categorical variable in three different models. In model 1, no covariates were adjusted. Model 2 was adjusted for gender, age, and race. Model 3 was adjusted for gender, age, race, education level, serum glucose, serum phosphorus, serum iron, serum calcium, serum CRP serum, serum 25 (OH) D, systolic blood pressure, diastolic blood pressure, body mass index, hypertension, diabetes, chronic kidney disease, protein intake, calcium intake, and phosphorus intake. To further explore the association between DII with PTH and HP in different population settings, the subgroup analysis was performed by stratified weighted multivariate regression analysis. In addition, it has been well-recognized that patients with CKD often have elevated parathyroid hormone levels; the CKD status was treated as a prespecified potential effect modifier. An interaction term was added to test the heterogeneity of associations between the subgroups. *P* < 0.05 was considered statistically significant. All analysis was performed using Empower software (www.empowerstats.com; X&Y solutions, Inc., Boston MA) and R version 3.4.3 (http://www.R-project.org, The R Foundation).

## Results

### Baseline Characteristics of Participants

The weighted demographic characteristics and other covariates of included individuals were shown in Table 1. A total of 7,679 participants were included in this study, of whom 48.34% were males and 51.66% were females, with an average age of 46.73 ± 18.80 years old. Median DII was −0.24 (−2.20 to 1.80), and the ranges of DII for tertiles 1–3 were −6.60 to −1.50, −1.50 to 1.08, and 1.08 to 6.50, respectively. Among different tertiles of DII, gender, race, education level, systolic blood pressure, diastolic blood pressure, serum iron, serum CRP, serum 25 (OH) D, urinary creatinine, serum creatinine, parathyroid hormone, and HP, whether having hypertension, diabetes, low eGFR and CKD, were significantly different, while no significant difference was observed in BMI, serum glucose, serum phosphorus, serum calcium, urinary albumin, eGFR, and whether having albuminuria between different tertiles. Mean PTH was 43.42 ± 23.21 pg/ml, and the average PTH was 45.53 ± 26.63 pg/ml for the participants in the highest tertile group compared with 41.42 ± 19.74 pg/ml in the lowest tertile group (*P* < 0.0001). The rate of HP was 11.15% overall, while the rate in the highest DII tertile was 13.28 and 8.60% in the lowest DII tertile (*P* < 0.0001).

**Table 1:** Weighted baseline characteristics of the participants according to different dietary inflammatory indexes (DIIs).

**DII**	**Overall**	**Tertile 1**	**Tertile 2**	**Tertile 3**	***P*-value**
	**−0.24 (-2.20**~**1.80)**	**(−6.60**~**-1.50)**	**(−1.50**~**1.08)**	**(1.08**~**6.50)**	
Age (years)	46.73 ± 18.80	45.59 ± 17.88	47.51 ± 19.13	47.26 ± 19.46	0.0003
Gender (%)					
Male	48.34	60.84	46.40	35.06	<0.0001
Female	51.66	39.16	53.60	64.94	
Race (%)					
Mexican American	12.36	12.42	13.10	11.45	0.0003
Other hispanic	3.18	2.92	3.18	3.50	
Non-hispanic white	64.84	66.83	65.22	61.95	
Non-hispanic black	14.77	13.25	13.98	17.53	
Others	4.86	4.58	4.52	5.58	
Education level (%)					
Less than high school	22.34	19.88	22.40	25.31	<0.0001
High school or general educational development	25.39	23.98	25.02	27.56	
Above high school	52.08	55.97	52.43	46.90	
Others	0.18	0.17	0.15	0.23	
Systolic blood pressure (mmHg)	122.59 ± 18.55	121.71 ± 16.48	123.43 ± 19.30	122.76 ± 20.03	0.0043
Diastolic blood pressure (mmHg)	70.18 ± 13.32	71.02 ± 12.86	69.60 ± 13.72	69.78 ± 13.39	0.0002
Body mass index (kg/m^2^)	28.20 ± 6.64	28.13 ± 6.47	28.35 ± 6.70	28.11 ± 6.77	0.3685
Glucose, serum (mg/dl)	96.83 ± 30.23	96.63 ± 28.77	96.91 ± 30.54	96.98 ± 31.61	0.9074
Phosphorus, serum (mg/dl)	3.84 ± 0.57	3.85 ± 0.57	3.84 ± 0.57	3.85 ± 0.58	0.7647
Iron, serum (μg/dl)	87.38 ± 36.47	91.77 ± 36.78	85.76 ± 35.00	83.77 ± 37.13	<0.0001
C-reactive protein, serum (mg/dl)	0.41 ± 0.83	0.33 ± 0.63	0.42 ± 0.80	0.48 ± 1.04	<0.0001
25(OH)D, serum (nmol/L)	59.96 ± 22.12	61.73 ± 21.49	60.02 ± 22.31	57.68 ± 22.46	<0.0001
Calcium, serum (mg/dl)	9.52 ± 0.35	9.53 ± 0.35	9.52 ± 0.35	9.51 ± 0.35	0.2978
Hypertension (%)					
Yes	29.58	25.26	32.51	31.71	<0.0001
No	70.42	74.74	67.49	68.29	
Diabetes (%)					
Yes	7.43	5.97	8.69	7.82	<0.0001
No	92.57	94.03	91.31	92.16	
Creatinine, urine (mg/dl)	127.53 ± 79.52	127.38 ± 75.49	123.34 ± 77.40	132.39 ± 86.15	0.0004
Albumin, urine (mg/L)	32.22 ± 225.38	28.30 ± 214.83	33.67 ± 230.78	35.44 ± 231.85	0.4891
Serum creatinine (mg/dl)	0.91 ± 0.29	0.94 ± 0.20	0.91 ± 0.36	0.89 ± 0.30	<0.0001
eGFR (ml/min/1.73 m^2^)	91.94 ± 23.47	91.93 ± 21.63	91.79 ± 23.75	92.14 ± 25.27	0.8722
Albuminuria (%)					
Yes	0.16	0.13	0.13	0.22	0.6457
No	99.84	99.87	99.87	99.78	
Low eGFR (eGFR <60 ml/min/1.73 m^2^, %)
Yes	8.28	6.34	8.64	10.27	<0.0001
No	91.72	93.66	91.36	89.73	
CKD (%)					
Yes	8.33	6.42	8.66	10.30	<0.0001
No	91.67	93.58	91.34	89.70	
Hyperparathyroidism (%)					
Yes	11.15	8.60	12.07	13.28	<0.0001
No	88.85	91.40	87.93	86.72	
Parathyroid hormone (pg/ml)	43.42 ± 23.21	41.42 ± 19.74	43.75 ± 23.29	45.53 ± 26.63	<0.0001

*eGFR, estimated glomerular filtration rate; CKD, chronic kidney disease*.

As for the PTH levels of the participants based on different renal conditions, the participants with albuminuria, low eGFR, and CKD tended to have higher PTH levels compared with their counterparts. In the CKD group, mean PTH was 68.40 ± 55.29 pg/ml for the highest tertile group while 51.12 ± 32.84 pg/ml for the lowest tertile group (*P* = 0.0002). Mean PTH was 68.45 ± 55.36 pg/ml for the highest tertile and 51.42 ± 32.91 pg/ml for the lowest tertile in the low eGFR group (*P* = 0.0003). In the albuminuria group, mean PTH was 131.86 ± 94.31 pg/ml for the highest tertile group while 95.06 ± 110.67 pg/ml for the lowest tertile group; however, there was no significant difference (*P* = 0.7248). Mean eGFR of the patients with CKD was 48.96 ± 10.64 ml/min/1.73 m^2^, and 1.78% of them were <15 ml/min/1.73 m^2^. We also calculated the mean PTH level according to different stages of CKD. The PTH level in the participants with CKD, stages 1 to 5, was 40.50 ± 18.45, 43.58 ± 19.97, 56.01 ± 32.16, 122.30 ± 81.21, and 239.12 ± 136.89 pg/ml. The participants tended to show a higher PTH level with the CKD progression (Table 2).

**Table 2:** Parathyroid hormone (PTH) and the prevalence of hyperparathyroidism (HP) based on different renal population settings, weighted.

		**DII Tertile** **1 (−6.60**~**-1.50)**	**DII Tertile** **2 (−1.50**~**1.08)**	**DII Tertile** **3 (1.08**~**6.50)**	***P* for trend**
Parathyroid hormone (pg/ml)					
Albuminuria					
Yes	111.73 ± 89.59	95.06 ± 110.67	99.18 ± 32.35	131.86 ± 94.31	0.7248
No	43.31 ± 22.80	41.35 ± 19.25	43.67 ± 23.19	45.34 ± 25.98	<0.0001
Low-eGFR^a^					
Yes	61.35 ± 45.69	51.42 ± 32.91	61.86 ± 41.57	68.45 ± 55.36	0.0003
No	41.80 ± 19.17	40.74 ± 18.31	42.03 ± 19.91	42.91 ± 19.31	0.0005
CKD^b^					
Yes	61.23 ± 45.62	51.12 ± 32.84	61.91 ± 41.53	68.40 ± 55.29	0.0002
Stage 1	40.50 ± 18.45	39.22 ± 17.38	40.39 ± 18.79	42.19 ± 19.18	<0.0001
Stage 2	43.58 ± 19.97	42.70 ± 19.27	44.29 ± 21.15	43.97 ± 19.44	0.1859
Stage 3	56.01 ± 32.16	48.81 ± 25.91	58.73 ± 36.05	59.15 ± 31.84	0.0011
Stage 4	122.30 ± 81.21	126.26 ± 84.37	93.96 ± 59.24	155.64 ± 89.70	0.0603
Stage 5	239.12 ± 136.89	75.00 ± 0.00	169.02 ± 124.24	265.17 ± 131.93	0.5695
No	41.80 ± 19.16	40.76 ± 18.31	42.02 ± 19.91	42.91 ± 19.31	0.0006
Hyperparathyroidism (%)					
Albuminuria					
Yes	54.86	26.34	67.48	66.91	0.0431
No	11.08	8.57	11.99	13.16	<0.0001
Low-eGFR^a^					
Yes	28.13	19.66	28.24	34.49	0.0018
No	9.62	7.85	10.54	10.85	0.0005
CKD^b^					
Yes	28.07	19.40	28.45	34.39	0.0014
Stage 1	8.88	7.14	8.78	11.08	0.0011
Stage 2	10.63	8.75	12.93	10.52	0.0127
Stage 3	24.87	17.78	25.53	29.86	0.0145
Stage 4	79.47	72.64	66.94	98.94	0.0400
Stage 5	78.57	0.00	42.71	90.54	0.1868
No	9.61	7.86	10.51	10.86	0.0005

*eGFR, estimated glomerular filtration rate; CKD, chronic kidney disease*.

a *Low eGFR was defined as eGFR <60 ml/min/1.73 m^2^*.

b *CKD stage 1: eGFR ≥ 90 ml/min/1.73 m^2^; stage 2: 60 ml/min/1.73 m^2^ ≤ eGFR <90 ml/min/1.73 m^2^; stage 3: 30 ml/min/1.73 m^2^ ≤ eGFR <60 ml/min/1.73 m^2^; stage 4: 15 ml/min/1.73 m^2^ ≤ eGFR <30 ml/min/1.73 m^2^; stage 5: eGFR <15 ml/min/1.73 m^2^*.

For the prevalence of HP, participants with reduced renal function tended to have higher rates of HP. In the population with CKD, 28.07% of the participants had HP, while it was 9.61% for those without CKD. Similar results could be observed in the albuminuria and low-eGFR population as well (Albuminuria: 54.86 vs. 11.08%, low eGFR: 28.13 vs. 9.62%) (Table 2).

### Higher DII Was Associated With Higher PTH Level and Higher Risk of HP

Weighted multivariable regression analysis was conducted to estimate the association of DII with the PTH level and HP in three different models (Table 3). The results revealed a positive association between DII scores and PTH with statistical significance (Model 1, β = 0.71, 95% CI: 0.51, 0.90, *P* < 0.0001; Model 2, β = 0.58, 95% CI: 0.38, 0.77, *P* < 0.0001; Model 3, β = 0.46, 95% CI: 0.25, 0.66, *P* ≤ 0.0001). According to the results of the fully adjusted model (Model 3), each unit of the increased DII score was associated with a PTH increase by.46 pg/ml, suggesting that the higher DII scores were associated with a higher PTH level. This association remained statistically significant after DII was grouped as tertiles. The fully adjusted effect size (reference to Tertile 1) was 1.56 (95% CI: 0.28, 2.84, *P* = 0.0167) for Tertile 2 and 2.66 (95% CI: 1.31, 4.01, *P* = 0.0001) for Tertile 3.

**Table 3:** Association between DII, PTH, and HP, weighted.

	**β/OR**^**a**^ **(95% CI**^**b**^**), P value**		
	**Model 1^c^**	**Model 2^d^**	**Model 3^e^**
Parathyroid hormone			
DII (continuous)	0.71 (0.51, 0.90) <0.0001	0.58 (0.38, 0.77) <0.0001	0.46 (0.25, 0.66) <0.0001
DII categories			
Tertile 1	Reference	Reference	Reference
Tertile 2	2.33 (1.09, 3.56) 0.0002	1.77 (0.54, 2.99) 0.0049	1.56 (0.28, 2.84) 0.0167
Tertile 3	4.11 (2.84, 5.39) <0.0001	3.31 (2.03, 4.60) <0.0001	2.66 (1.31, 4.01) 0.0001
Hyperparathyroidism			
DII (continuous)	1.07 (1.05, 1.10) <0.0001	1.07 (1.04, 1.09) <0.0001	1.05 (1.02, 1.08) 0.0023
DII categories			
Tertile 1	Reference	Reference	Reference
Tertile 2	1.48 (1.26, 1.75) <0.0001	1.43 (1.21, 1.68) <0.0001	1.27 (1.05, 1.55) 0.0157
Tertile 3	1.57 (1.33, 1.85) <0.0001	1.49 (1.26, 1.76) <0.0001	1.32 (1.09, 1.61) 0.0054

*Insensitivity analysis—dietary inflammatory index was converted from a continuous variable to a categorical variable (tertiles)*.

a *β: effect sizes; OR: odds ratio*.

b *95% CI: 95% confidence interval*.

c *Model 1: no covariates were adjusted*.

d *Model 2: adjusted for gender, age, and race*.

e *Model 3: adjusted for gender, age, race, education level, serum glucose, serum calcium, serum phosphorus, serum iron, serum C-reactive protein serum, serum 25(OH)D, systolic blood pressure, diastolic blood pressure, body mass index, hypertension, diabetes, chronic kidney disease, protein intake, calcium intake, and phosphorus intake*.

In terms of HP, we also observed that increased DII was associated with a higher risk of HP (Model 1, OR = 1.07, 95% CI: 1.05, 1.10, *P* < 0.0001; Model 2, OR = 1.07, 95% CI: 1.04, 1.09, *P* < 0.0001; Model 3, OR = 1.05, 95% CI: 1.02, 1.08, *P* = 0.0023). In Model 3, which adjusted for all covariates, the results indicated that each unit of increased DII score was associated with a 5% increase of risk of HP. In sensitivity analysis, the adjusted OR (reference to Tertile 1) was 1.27 (95% CI: 1.05, 1.55, *P* = 0.0157) for Tertile 2 and 1.32 (95% CI: 1.09, 1.61, *P* = 0.0054) for Tertile 3, suggesting a stable positive relationship between increased DII and higher risk of HP with statistical significance.

### Subgroup Analysis

We conducted the subgroup analysis stratified by low eGFR, CKD, gender, age, BMI, hypertension, and diabetes to further explore the association of DII with the PTH level and HP in different population settings by stratified weighted multivariate regression analysis and tested the interactions (Table 4). Regarding the correlation between DII scores and the PTH level, the test for interaction was significant for low eGFR (P for interaction = 0.0004) and CKD *P* (for interaction = 0.0003), indicating significant dependence on renal function. However, the positive association was statistically significant both in subgroups stratified by low eGFR and in subgroups stratified by CKD (all P for trend < 0.05). In subgroups stratified by gender, age, BMI, hypertension, and diabetes, the positive association between DII and PTH was still significant (*P* for trend <0.05) and P for interaction >0.05, suggesting that the correlation between DII scores and the PTH level was similar in the population with different gender, age, BMI, hypertension status, and diabetes status.

**Table 4:** Subgroup analysis stratified by different variables, weighted.

**DII**	**Parathyroid hormone**		**Hyperparathyroidism**	
	**β^a^ (95% CI^b^)**,	**P for interaction**	**OR^c^ (95% CI), *P* for trend**	
	***P* for trend**			***P* for interaction**
Low-eGFR^d^				
Yes	1.65 (0.30, 3.01) 0.0171	0.0004	1.10 (1.04, 1.17) 0.0018	0.7108
No	0.33 (0.15, 0.51) 0.0003		1.04 (1.01, 1.08) 0.0121	
CKD					
Yes	1.73 (0.39, 3.08) 0.0116	0.0003	1.07 (1.01, 1.16) 0.0023	0.4942
No	0.33 (0.15, 0.50) 0.0003		1.04 (1.01, 1.08) 0.0121	
Gender				
Male	0.47 (0.20, 0.74) 0.0006	0.8715	1.04 (1.00, 1.09) 0.0469	0.5675
Female	0.43 (0.12, 0.73) 0.0061		1.05 (1.01, 1.10) 0.0206	
Age				
<60 years old	0.32 (0.10, 0.53) 0.0038	0.0588	1.04 (1.00, 1.08) 0.0444	0.5734
≥60 years old	0.89 (0.41, 1.37) 0.0003		1.07 (1.01, 1.12) 0.0137	
BMI				
BMI <25 kg/m^2^	0.48 (0.15, 0.81) 0.0042	0.7475	1.11 (1.05, 1.17) 0.0004	0.1499
BMI ≥ 25 kg/m^2^	0.47 (0.21, 0.73) 0.0003		1.02 (1.01, 1.06) 0.0039	
Hypertension				
Yes	0.69 (0.21, 1.17) 0.0053	0.1696	1.04 (0.99, 1.09) 0.0862	0.7086
No	0.34 (0.14, 0.55) 0.0009		1.05 (1.01, 1.09) 0.0071	
Diabetes				
Yes	1.09 (0.12, 2.06) 0.0287	0.1881	1.09 (0.98, 1.21) 0.1276	0.3318
No	0.40 (0.20, 0.61) 0.0001		1.04 (1.01, 1.08) 0.0035	

*The results show that the subgroup analysis was adjusted for all presented covariates except the effect modifier. eGFR, estimated glomerular filtration rate; CKD, chronic kidney disease*.

a *β: effect sizes*.

b *95% CI: 95% confidence interval*.

c *OR: odd ratio*.

d *Low eGFR was defined as eGFR <60 ml/min/1.73 m^2^*.

As for the association between DII scores and HP, results of subgroup analysis stratified by CKD or no CKD demonstrated that DII was positively associated with a higher risk of HP (OR = 1.07, *P* for trend = 0.0023) in the CKD subgroup, and a similar result (OR = 1.04, *P* for trend = 0.0121) was observed in the non-CKD group. In addition, an interaction test was performed to evaluate if there was any significant dependence of the effect modifier (CKD) on the association. *P* for interaction >0.05 means no significant dependence, indicating that the magnitude of the association was the same for the population with/without CKD (*P* for interaction = 0.4942). Similarly, we did not find any significant dependence on low eGFR, gender, age, BMI, hypertension status, and diabetes status (all *P* for interaction > 0.05). These results indicated that the positive association between DII scores and HP was similar in the population with different renal function conditions (including CKD and low eGFR), gender, age, BMI, hypertension status, and diabetes status and could also be appropriate for the participants with reduced renal function.

## Discussion

In this cross-sectional study with 7,679 adults, a significant positive association of DII with the PTH level and HP was observed, indicating that higher consumption of pro-inflammatory diet may contribute to the higher PTH level and an increased risk of HP. The association between the exposure variable and outcome variables was still stable after adjustment for covariates. Subgroup analysis stratified by the renal condition (low eGFR and CKD) and other variables showed that this positive association was not affected, suggesting that this association could be appropriate for the population with different renal function conditions, gender, age, BMI, hypertension status, and diabetes status.

To our knowledge, this is the first study that assesses the association between the dietary inflammatory potential with the PTH level and the risk of HP. Patel et al. (44) investigated whether dietary calcium intake will influence the serum PTH concentrations in apparently healthy Indian adolescents and found that subjects with higher calcium intake had lower PTH. Cheung et al. (45) suggested that low dietary magnesium intake could alter the vitamin D-PTH relationship in adults who were overweight or obese. In another study assessing the effect of the dietary approaches to stop hypertension (DASH) diet, which is rich in fiber and low-fat dairy and is useful for lowering blood pressure on the PTH level, no significant effect was observed (46). Additionally, the cause-and-effect relationship between PTH and inflammation still remains controversial. Cheng et al. (19) assessed the association between the PTH level and inflammatory markers among the U.S. adults and reported that higher inflammatory markers were associated with a higher PTH level. Similar results have been reported before in other studies (22, 47–49). Another study observed upregulation of inflammatory genes in the adipose tissue from primary patients with HP when compared with the healthy controls (19). However, conflicting results were found for the effects of inflammatory markers on patients with HP after parathyroidectomy: both increased (24, 50, 51) and decreased (22) and even unchanged levels of inflammatory markers have been reported (52). The inconsistent results indicated an unclear cause-and-effect association between PTH and inflammation. The results of this study indicated that higher pro-inflammatory dietary intake was positively associated with PTH and an increased risk of HP; in another way, a higher inflammatory level may be associated with a higher PTH level and a higher risk of HP. The association was similar in the population with different renal function conditions, gender, age, BMI, hypertension status, and diabetes status according to the subgroup analysis.

Regarding the positive association between DII scores and the PTH level, we observed significant dependence on low eGFR (P for interaction = 0.0004) and CKD (P for interaction = 0.0003), indicating that renal function may be affected by this association. Serious disorders of calcium and phosphorus metabolism were commonly observed in patients with CKD; we speculated that the effect of the pro-inflammatory diet on PTH may be interfered by the metabolism disorder (53). The interplay between Renin-Angiotensin-Aldosterone System (RAAS) and PTH may also influence this association (54). What is more, patients with end-stage CKD were treated with hemodialysis usually, and the management of hemodialysis can affect the calcium and phosphorus metabolic status (55, 56). However, the data of hemodialysis in NHANES were missing, so we could not exclude the influence of different dialysis modes or conduct further analysis. Additionally, it was worth noting that this positive association was still statistically significant both in subgroups stratified by low eGFR and CKD (all *P*-value <0.05), suggesting that, although, renal function may affect this association, it was similar in the population with/without low eGFR and CKD.

The exact mechanism of this positive association of DII with PTH and HP remains unclear. A possible explanation to support the results might be the effect of diet on pro-inflammatory markers, such as IL-8, TNF-α, etc., Previous studies have demonstrated that inflammation was closely associated with the activation of the NF-κB pathway (57–59). NF-κB could serve as a direct regulator of genes related to cell proliferation, such as cyclin D1, p21, p27, and p53, thus, involved in cell cycles (60, 61). It also plays a key role in angiogenesis and tumor growth by promoting antiapoptotic mechanisms and the expression of inflammatory cytokines; in turn, these cytokines also contribute to its activation (62, 63). NF-κB could play a role in PTH regulation as well. Mao et al. (26) found that local NF-κB activation could promote the synthesis and secretion of PTH and mediated the transcriptional activation of PTH directly. We hypothesized that the elevated inflammatory level may lead to the activation of NF-κB; thus, similar regulation by NF-κB may underlie the development of parathyroid hyperplasia and enhanced PTH synthesis and secretion. Angeletti et al. (27) evaluated the effects of IL-8 on the PTH level by incubation of parathyroid cells with recombinant IL-8 *in vitro* and found that IL-8 increased both PTH secretion and PTH mRNA expression. A previous study also reported that TNF-α could affect CASR transcription *via* NF-κB in human renal tubular cells (64). A significant down-expression of CASR mRNA, thus, upsetting the Ca^2+^ set point and enhancing the sensitivity of parathyroid glands to Ca^2+^ concentration may be another potential explanation of our results.

One of the strengths of our study was that it was based on nationwide, population-based sampling survey data, and sample weights were adopted, which make the study much more representative. It is noteworthy that we performed subgroup analysis stratified by different renal functions and found that this association was similar in different population settings and could also be appropriate for the participants with CKD. However, the limitations of this study cannot be ignored. First, due to the cross-sectional study design, we cannot make a causal inference. Second, the food intake data were based on a 24-h dietary recall; a recall bias was inevitable, and the daily variability of food intake cannot be reflected. With regard to DII scores, a total of 45 food parameters were included according to the design, but only 27 food parameters were available in the NHANES data. Although, previous studies reported that the predictive ability was not affected when <30 dietary parameters were used with DII calculating (38–40), the impact on accuracy cannot be ignored. Third, HP was defined as intact PTH > 65 pg/ml in our analysis. However, optimal PTH levels in CKD stages 3–5 remain unknown (65). Although most of the participants with CKD in this study were in stage 1 and stage 2, we also used this HP definition in patients with CKD stages 3–5; thus, it may affect the accuracy. In addition, some potential confounders, such as the hemodialysis condition (the patients with non-dialysis dependent or hemodialysis CKD, a hemodialysis type, etc.,), drugs use, and fibroblast growth factor 23 (FGF23), may influence this association, but these confounders were not available in NHANES data (56, 66–68). Another limitation was that PTH was only assayed at a single time point; thus, no repeat measurements of PTH were conducted.

## Conclusion

In this cross-sectional study with 7,679 adults, a significant positive association between DII with PTH and HP was observed, indicating that higher consumption of pro-inflammatory potential correlates positively with the higher PTH level and an increased risk of HP. This association exists both in the populations with CKD and with no CKD. Our finding suggests that anti-inflammatory dietary management may reduce the risk of HP. Further research and clinical settings are still needed to validate their potential application.

## Data Availability Statement

Publicly available datasets were analyzed in this study. This data can be found here: www.cdc.gov/nchs/nhanes/.

## Ethics Statement

The studies involving human participants were reviewed and approved by the NCHS Ethic Review Board. The patients/participants provided their written informed consent to participate in this study.

## Author Contributions

ZQ: data analysis, software, and writing of the original draft. QY: formal analysis and software. RL: methodology and writing of the original draft. BS: conceptualization, funding acquisition, writing, reviewing, and editing. All the authors approved the final version.

## Conflict of Interest

The authors declare that the research was conducted in the absence of any commercial or financial relationships that could be construed as a potential conflict of interest.
